# Stretchable, Transparent, and Ultra-Broadband Terahertz Shielding Thin Films Based on Wrinkled MXene Architectures

**DOI:** 10.1007/s40820-024-01365-w

**Published:** 2024-04-02

**Authors:** Shaodian Yang, Zhiqiang Lin, Ximiao Wang, Junhua Huang, Rongliang Yang, Zibo Chen, Yi Jia, Zhiping Zeng, Zhaolong Cao, Hongjia Zhu, Yougen Hu, Enen Li, Huanjun Chen, Tianwu Wang, Shaozhi Deng, Xuchun Gui

**Affiliations:** 1 State Key Laboratory of Optoelectronic Materials and Technologies, School of Electronics and Information Technology, Sun Yat-Sen University, Guangzhou, 510275 People’s Republic of China; 2 National Key Laboratory of Materials for Integrated Circuits, Shenzhen Institute of Advanced Electronic Materials, Shenzhen Institute of Advanced Technology, Chinese Academy of Sciences, Shenzhen, 518055 People’s Republic of China; 3 Guangdong Province Key Laboratory of Display Material and Technology, Guangzhou, 510275 People’s Republic of China; 4 China Academy of Aerospace Science and Innovation, Beijing, 100176 People’s Republic of China; 5 School of Materials Science and Engineering, Sun Yat-Sen University, Guangzhou, 510275 People’s Republic of China; 6 GBA Branch of Aerospace Information Research Institute, Chinese Academy of Sciences, Guangzhou, 510700 People’s Republic of China; 7 School of Electronic, Electrical and Communication Engineering, University of Chinese Academy of Sciences, Beijing, 100049 People’s Republic of China; 8 Guangdong Provincial Key Laboratory of Terahertz Quantum Electromagnetics, Guangzhou, 510700 People’s Republic of China

**Keywords:** Terahertz, Wrinkle structure, Electromagnetic interference shielding, MXene

## Abstract

With the increasing demand for terahertz (THz) technology in security inspection, medical imaging, and flexible electronics, there is a significant need for stretchable and transparent THz electromagnetic interference (EMI) shielding materials. Existing EMI shielding materials, like opaque metals and carbon-based films, face challenges in achieving both high transparency and high shielding efficiency (SE). Here, a wrinkled structure strategy was proposed to construct ultra-thin, stretchable, and transparent terahertz shielding MXene films, which possesses both isotropous wrinkles (height about 50 nm) and periodic wrinkles (height about 500 nm). Compared to flat film, the wrinkled MXene film (8 nm) demonstrates a remarkable 36.5% increase in SE within the THz band. The wrinkled MXene film exhibits an EMI SE of 21.1 dB at the thickness of 100 nm, and an average EMI SE/t of 700 dB μm^−1^ over the 0.1–10 THz. Theoretical calculations suggest that the wrinkled structure enhances the film's conductivity and surface plasmon resonances, resulting in an improved THz wave absorption. Additionally, the wrinkled structure enhances the MXene films' stretchability and stability. After bending and stretching (at 30% strain) cycles, the average THz transmittance of the wrinkled film is only 0.5% and 2.4%, respectively. The outstanding performances of the wrinkled MXene film make it a promising THz electromagnetic shielding materials for future smart windows and wearable electronics.

**Supplementary Information:**

The online version contains supplementary material available at 10.1007/s40820-024-01365-w.

## Highlights

- A stretchable, transparent, and ultra-broadband (0.1–10 THz) terahertz shielding MXene (Ti_3_C_2_T_x_) film has been fabricated by a structure engineering strategy.
- Theoretical calculations indicate that the wrinkled structure enhances the film's conductivity and surface plasmon resonances, resulting in an improved THz wave absorption.
- The wrinkled MXene films exhibit superb conformability to surfaces with random curvatures, and can be used as a shielding film for THz imaging.

**Supplementary Information:**

The online version contains supplementary material available at 10.1007/s40820-024-01365-w.

## Graphical abstract

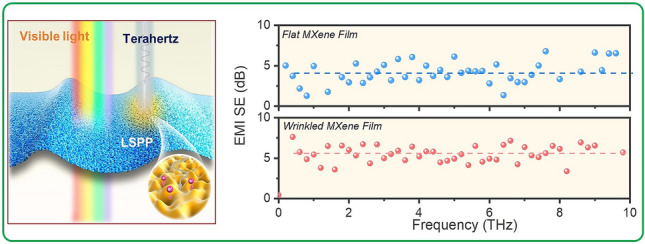

## Introduction

Terahertz (THz) waves (0.1–10 THz) exhibit unique broadband, fingerprint spectrum, and transient characteristics [1–6]. As THz technology rapidly advances in security inspection, information communication, and intelligent electronics, concerns over security risks, such as electromagnetic radiation, interference, and information leakage, have grown more significantly [7–12]. Consequently, the development of THz electromagnetic interference (EMI) shielding materials becomes crucial for ensuring electromagnetic protection and information confidentiality [6, 7, 13]. Moreover, in the aforementioned applications, the shielding materials also need to have good light transmittance, flexibility, and surface conformability to be suitable for flexible terahertz electronic devices. According to Schelkunoff's theory [14], the EMI shielding efficiency (EMI SE) is closely related to the conductivity of thin film-type materials. Although traditional metal shielding materials have ultra-high conductivity, their opacity, low flexibility, and limited stretchability present challenges in meeting the above requirements [15–20]. Therefore, there is an urgent need for new materials or strategies to resolve the conflict between small thickness, high transmittance, and flexibility, while maintaining high EMI SE.

Recently, it has been discovered that two-dimensional (2D) materials, such as MXene and graphene, possess strong absorption capabilities, and high THz wave shielding efficiency, which are associated with their abundant surface groups and high conductivity [21–23]. In particular, MXenes, a class of 2D materials, possess exceptional electrical conductivity and mechanical flexibility, making them suitable candidates for THz shielding applications [24–26]. Different from other 2D materials (such as graphene, h-BN or TMD), Ti_3_C_2_T_x_ nanosheets with high intrinsic electrical conductivity exhibit weak coupling effects and almost independently polarized entities [27]. The weak interaction between nanosheets can induce surface plasmon resonance and promote the absorption of electromagnetic waves. For instance, a MXene film with a thickness of 12 μm demonstrates an impressive EMI SE of up to 17.0 dB for THz waves [28]. The nanometer-thick MXene film shields about 70% of THz waves, including nearly 50% of incident electromagnetic waves being absorbed [26]. However, achieving a balance between transparency and EMI shielding performance in MXene films proves to be a big challenge. This is primarily due to the reflection loss shielding mechanism in MXene films, which is a result of their high conductivity [29]. While reducing the thickness of MXene films can enhance their transmittance, it also leads to a significant decrease in the reflection loss, thereby reducing the EMI SE [30]. For instance, a 20 nm thick MXene film achieves a 90% transmittance, but only has an EMI SE value of 2.5 dB [31, 32]. Recently, structuring of the thin film presents an effective approach to enhance their electromagnetic responses [33–37]. For example, previous studies indicate that structuring a conductive film into periodic architectures can significantly amplify its local surface plasmon resonances (LSPRs) and facilitate the absorption of electromagnetic waves, particularly in the infrared and visible light spectrum [38]. Moreover, the periodic structures can also improve the mechanical stability of the films. However, this improvement usually comes at the expense of sacrificing the transparency of the thin films.

**Fig. 1:**
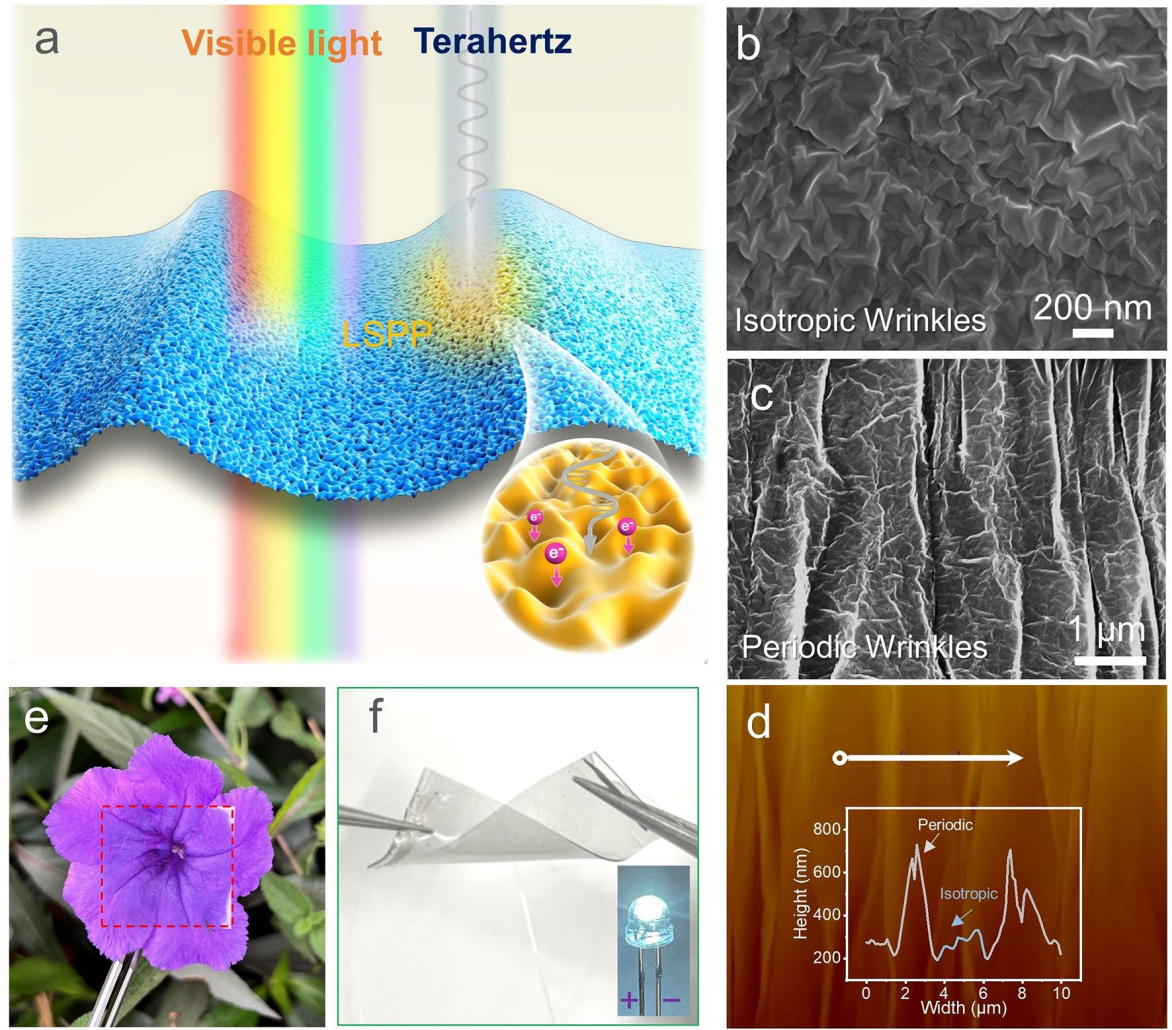
Structural and micromorphology characterization of the Ti_3_C_2_T_x_ MXene films. **a** Schematic diagram of the wrinkled film, where the visible light can be transmitted, but terahertz waves are effectively isolated. The wrinkled structure results in the enhancement of local SPP, thereby enhancing its absorption of terahertz electromagnetic waves. SEM images of **b** wrinkle-I film, and **c** wrinkle-P film. **d** AFM image of wrinkle-P film. **e** Digital photo of a wrinkle-I film on a flower. **f** A wrinkle-I film twisted at the ends. Inset, a lighting LED connected by the twisted wrinkle-I film

Here, we establish a structure engineering strategy to fabricate a transparent, flexible, stretchable, and highly effective THz EMI shielding MXene (Ti_3_C_2_T_x_) film, with an atomic thickness of only 8 nm, achieved through the formation of a wrinkled structure (Fig. 1a). The fabrication of the wrinkled MXene film was accomplished using an interfacial self-assembly method [39]. The incorporation of the wrinkled structures led to a remarkable increase of 36.5% in the EMI SE for THz waves (up to 5.6 dB) of the film, while it caused only a marginal decrease of 3.6% in its transmittance for visible light. Theoretical calculations unveil that the wrinkled structure enhances the conductivity and LSPRs of the MXene film, thereby boosting its absorption of THz waves. Furthermore, the incorporation of wrinkled structures significantly enhances the stretchability and structural stability of the films, resulting in a consistently low THz transmittance (< 2.5%) even when subjected to cyclic stretching and bending. Moreover, the MXene films with wrinkled structures exhibit superb conformability to surfaces with random curvatures, allowing for high-resolution THz imaging shielding of metals, leaves, and bamboo, etc.

## Experimental Methods

### Preparation of Ti_3_C_2_T_x_ Nanosheets

Ti_3_C_2_T_x_ nanosheets were prepared using conventional etching methods, as reported by our group previously [28]. A mixed solution of LiF and HCl was utilized to etch the Al layer from the Ti_3_AlC_2_ (MAX) phase. The obtained dark-green Ti_3_C_2_T_x_ suspension was then sonicated and centrifuged at 8000 rpm, and then frozen to obtain Ti_3_C_2_T_x_ nanosheets in the form of powder.

### Preparation of Transparent, Flat Ti_3_C_2_T_x_ Film and Wrinkle-I Ti_3_C_2_T_x_ Film

A total of 160 mg of Ti_3_C_2_T_x_ powders were dispersed in 20 mL of deionized water and sonicated for 10 min to obtain a Ti_3_C_2_T_x_ dispersion. To enhance the surface hydrophilicity of the substrate, they were treated in plasma for 7 min. Then, the Ti_3_C_2_T_x_ dispersion was spin-coated on the quartz sheet (or silicon wafer) at 1000 rpm to obtain a flat film. Under the same preparation parameters, wrinkle-I film was prepared using PDMS as the substrate. By repeatedly spin coating on a substrate with the same process parameters, flat film or wrinkled-I film with different thicknesses can be obtained.

### Preparation of Wrinkle-P Film

The wrinkle-P films were obtained through an interfacial self-assembly method. First, 40 mg of Ti_3_C_2_T_x_ powder was dispersed in 5 mL of deionized water and sonicated for 1 min. Then, 15 mL of ethanol was added to the Ti_3_C_2_T_x_ nanosheet solution and mixed evenly. 20 mL of the obtained solution was then dropped onto the surface of deionized water. The nanosheets were self-assembled into a continuous MXene film in the solution-air interface, as reported in our early work [39]. Second, a hydrophilized PDMS (with a thickness of 200 μm) substrate was pre-stretched on a customized manual displacement stage with set strains. The continuous MXene film was transferred film onto the pre-stretched PDMS substrate by wet transfer. Finally, by releasing the pre-strain in the PDMS, a wrinkle-P film was obtained. The thickness of the wrinkle-P films can be controlled by the volume of the solution dropped on the surface of deionized water.

### Characterization

The thickness of the film and the height of the winkles were measured via atomic force microscopy (AFM, Bruker, Dimension Icon). The morphology of the wrinkled film was observed using field-emission scanning electron microscopy (FE-SEM, Hitachi, S-4800) with a customized manual displacement stage. The transmittance spectra of films were measured via a UV–vis spectrophotometer (UV-2700, Shimadzu, Japan). X-ray diffraction (XRD, Bruker, D8 ADVANCE) and X-ray photoelectron spectroscopy (XPS, Thermo Scientific, ESCALAB 250Xi) were carried out to analyze the structure characters of the films. The EMI SE was conducted in the X-band using a network analyzer (N5232, VNA, Keysight), and in the terahertz band by a fiber-coupled terahertz time-domain spectroscopy (THz-TDS) system (TDS, BATOP, TDS 1008). Broadband Terahertz spectroscopy up to 10 THz was performed using two-color laser induced air plasma terahertz generation system combined with air biased coherent detection method. The flat films on quartz sheet (or high resistance silicon), and wrinkled films on the PDMS were used for THz testing.

## Results and Discussion

### Fabrication and Characterization of the Ti_3_C_2_T_x_ Films

The Ti_3_C_2_T_x_ MXene nanosheet was fabricated using traditional etching methods [40]. It consists of single and few-layer nanosheets with a thickness of 2 nm and a lateral size of 1–5 μm (Fig. S1). The large size of the nanosheets is beneficial for improving the mechanical and electrical properties of the self-assembled MXene film. The X-Ray diffraction (XRD) and Raman spectra confirmed the successful fabrication of Ti_3_C_2_T_x_ nanosheets (Fig. S2a, b). The XPS survey spectra showed that the MXene nanosheets contain a large number of oxygenic functional groups, which facilitated the self-assembly process of the film (Fig. S2c). The nanosheets are used to fabricate films on different substrates, such as quartz sheet and PDMS, by spin coating or interface self-assembly methods. The film assembled on a quartz sheet is continuous and uniform, without any voids, cracks, and ripples, even though its thickness is only 8 nm (Fig. S2d-f). In this work, the sample prepared on a quartz sheet (or silicon wafer) is defined as flat film. The thickness of the film can be controlled by spin coating times (Fig. S3). The transmittance of the flat film (8 nm) is about 75.4% (@550 nm) (Fig. S4a). Interestingly, using the same spin coating process but using PDMS as the substrate, the surface of the prepared MXene film (named as wrinkle-I film) shows an isotropous and homogeneous wrinkled structure with a wrinkle height of about 50 nm (Fig. 1b). The formation of these wrinkled structures may be attributed to the internal stress of the MXene film on the PDMS [41]. According to early reports, the wrinkled structures in the 2D materials based-film can improve its deformability [39]. To further increase the size of wrinkles, we prepared a wrinkled MXene film with a longitudinal periodic wrinkled structure (named as wrinkle-P film) by using a pre-stretched PDMS substrate. This film has both isotropic small-size winkles and periodic large wrinkles with a height of about 500 nm (Fig. 1c). The AFM image has clearly exhibited periodic wrinkles with longitudinal distribution (Fig. 1d). At the same time, the AFM height profiles further demonstrate that the height of the longitudinal wrinkles is about 500 nm, and that of the isotropous wrinkles is about 50 nm. Although wrinkles are formed in the film, it has almost no effect on its transmittance. The transmittance of the wrinkle-I film is still as high as 71.8% (Fig. S5), allowing for clear visibility of a flower (Fig. 1e). Furthermore, the transparent wrinkled MXene film also shows high conductivity and excellent flexibility, which can be used as a flexible conductor for lighting a LED bulb (Fig. 1f).

### THz EMI Shielding Performances of the Ti_3_C_2_T_x_ Films

**Fig. 2:**
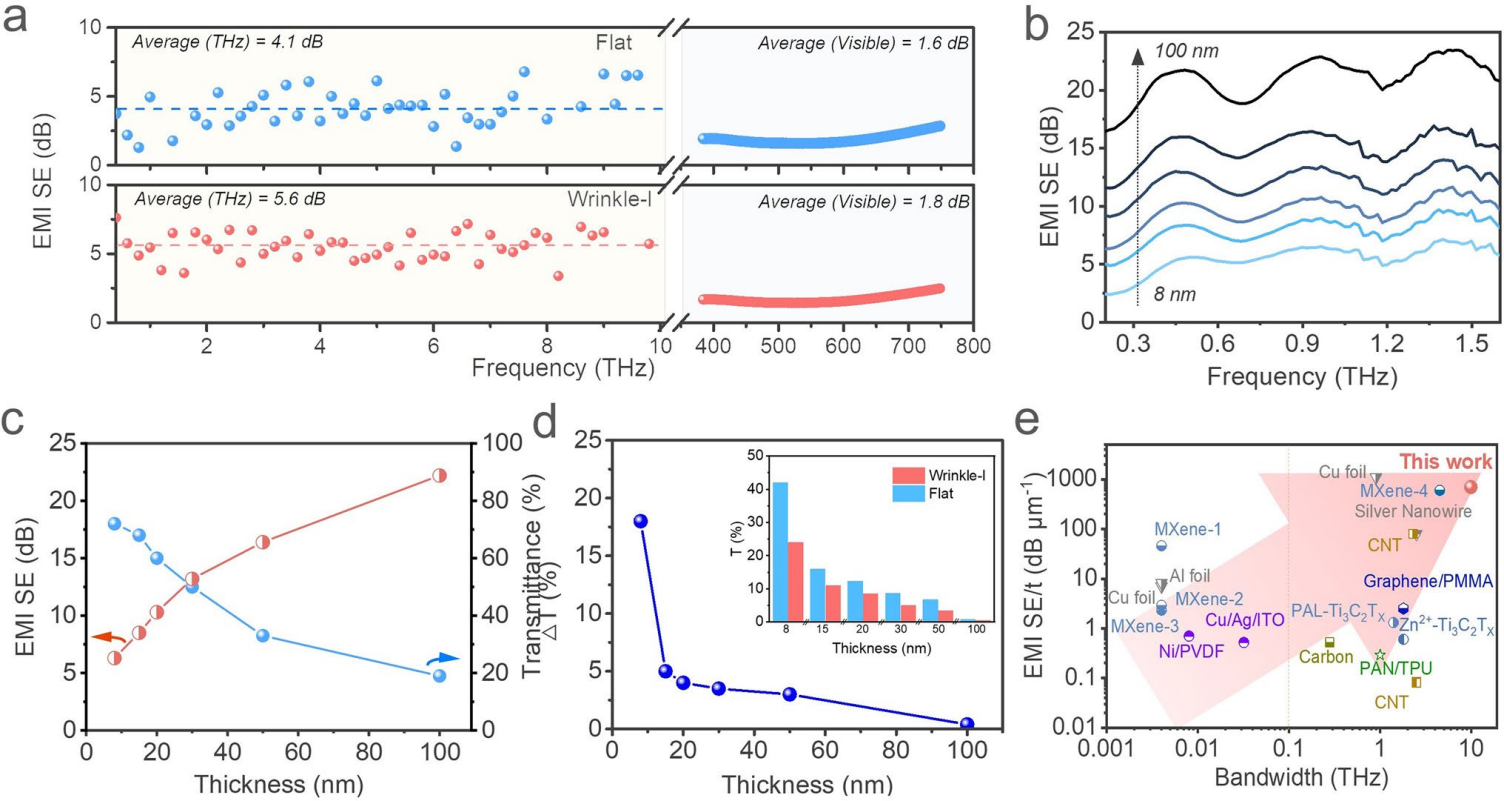
THz wave shielding performance of the MXene film. **a** EMI SE of wrinkle-I film and flat film in terahertz (0.1–10.0 THz) and visible light (400–780 nm, corresponding to 750–384 THz) bands. **b** EMI SE of wrinkle-I films with different thicknesses in a frequency range of 0.2–1.6 THz. **c** EMI SE (@1.0 THz) and visible light transmittance (@550 nm) of wrinkle-I films with different thicknesses. **d** Transmittance difference (△T) between wrinkle-I and flat films with different thicknesses. Inset: the transmittance of wrinkle-I and flat films at 1.0 THz. **e** Comparison of the EMI SE/t versus bandwidth between wrinkle-I film and other THz shielding materials (detailed data thereof are listed in Table S1†)

A Terahertz time-domain spectroscopy (THz-TDs) was employed to measure the EMI SE and transmission properties of MXene films in the THz band (Fig. S6). Figure 2a shows the total EMI SE (SE_T_) of wrinkle-I film and flat film with a thickness of 8 nm in the THz band (0.1 -10 THz) and visible spectrum (400–780 nm). The SE_T_ of the wrinkled-I film reached approximately 5.6 dB (corresponding to a transmittance of 27.5%), about 36.5% higher than the value of the flat film (4.1 dB). Interestingly, the visible light transmittance of the wrinkled film was only 3.6% lower than that of flat film at 550 nm. This difference in SE between the THz band and visual spectrum indicated that the wrinkled structures endow the MXene film with selective-shielding features. More details of transmission for the wrinkled-I films with different thicknesses in the THz band are shown in Figs. S7 and S8. We believe that the reason for this enhancement may be because the winkled structure not only enhances its conductivity but also causes changes in the electromagnetic response characteristics of the MXene film surface. A detailed analysis will be discussed in the following text. The EMI SE of the wrinkled film also increased with its thickness (Figs. 2b and S9) due to the increase in conductivity of MXene films (Fig. S4b). When the thickness of the wrinkle-I film is 100 nm, its EMI SE can reach up to 21.1 dB (99% of THz EM waves are shielded), which meets the commercial requirement of EMI shielding materials. In the EMI SE curves, there are three peaks at specific frequencies (0.5, 1.0, and 1.5 THz), which may be attributed to the Fabry–Pérot resonance effect between the wrinkle-I film and PDMS substrate.

In order to clearly compare the transmittance of the wrinkle-I film and flat films, the 1.0 THz was chosen as the contrasting frequency. Figure 2c shows the thickness-dependent THz EMI SE (@1.0 THz) and optical transmittance (@550 nm) characteristics, which vary almost linearly with thickness. In order to analyze the EMI performance of samples, the transmittances of wrinkle-I and flat films at 1.0 THz were compared (inset of Fig. 2d). The results indicate that as the thickness of the film decreases, the transmittance difference (△T_THz_) between wrinkle-I and flat films becomes larger (Fig. 2d), indicating that at lower thicknesses, the wrinkled structure in the MXene film will result in lower transmittance and higher EMI SE of the sample. The transmittance difference between the two films is greater than 17.5% when the film thickness is less than 10 nm. Therefore, for an ultra-thin film, forming microstructures such as wrinkles on its surface is a feasible method to improve its EMI SE for THz band without reducing its transparency. In order to more objectively evaluate the films’ performance, the specific shielding efficiency (SE/t) values of the wrinkled MXene film in this work were compared with those of other THz EMI shielding materials (Fig. 2e and Table S1) [15, 25, 42–50]. Obviously, the wrinkled film in this work has better EMI SE/t and wider bandwidth. Many thin film materials, such as metal foils, CNTs films, and graphene films, have been used for THz wave electromagnetic shielding. However, their shielding bandwidths are relatively limited, typically less than 2.0 THz. In contrast, the wrinkled MXene film with a thickness of 8 nm had an average EMI SE/t of 700 dB μm^−1^ over the 0.1–10 THz.

**Fig. 3:**
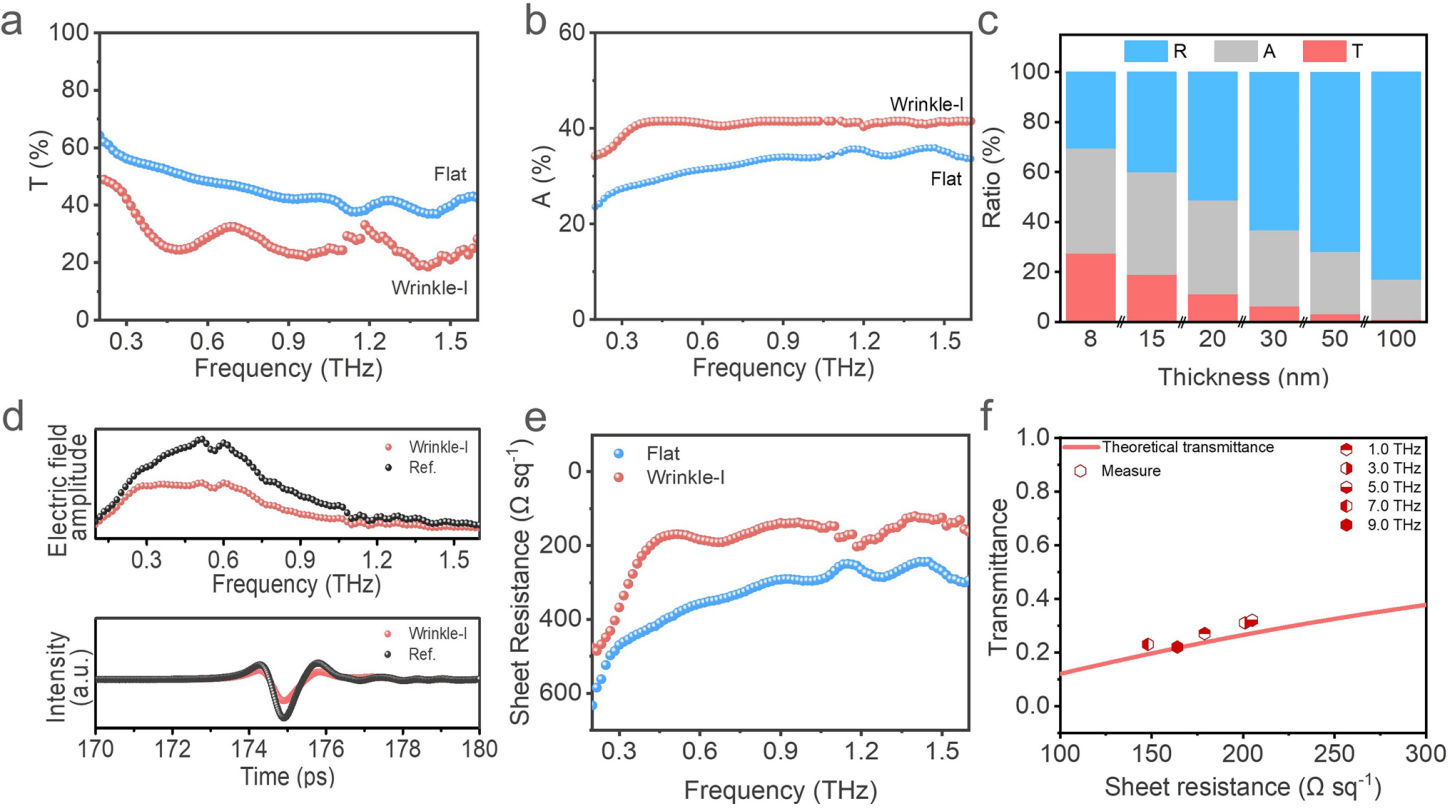
Transmittance, absorption, and mechanism of the MXene films for THz waves. **a** Transmittance, and **b** absorption of wrinkle-I and flat films in a frequency range of 0.2–1.6 THz. **c** Ratio of transmission, absorption, and reflection of wrinkle-I films with different thicknesses. **d** Transmission amplitude spectra (up) of the wrinkle-I film, obtained by Fourier transforming the transients (down). **e** Terahertz sheet resistances of the films in 0.2–1.6 THz. **f** A comparison of theoretical (Woltersdoff equations) and experimental transmittance for wrinkle-I films (8 nm) at different frequency points

To further understand the effect of wrinkled structure on the shielding feature of the film, the transmittance, reflection, and absorption of the wrinkled film in the frequency range of 0.2–1.6 THz were systematically calculated and analyzed. As shown in Fig. 3a, wrinkle-I films (8 nm) have a lower THz transmittance compared to the flat films. Its transmittance is about 27.5%, which means that about 72.5% of the incident THz electromagnetic waves are shielded. In comparison, only about 61.2% of the incident electromagnetic waves were shielded for the flat film with the same thickness. We found that the decrease in THz wave transmittance of wrinkled films is mainly due to the increased absorption (Fig. 3b). The average absorption of the wrinkle-I film is about 42.0%, while the absorption of the flat film is only 32.4% (Fig. S9). Moreover, as the thickness of the film increases from 8 to 100 nm, the absorption ratio gradually decreases from 42.0% to 16.0%, and the reflection ratio increases from 30.5% to 83.0% for the wrinkle-I film (Fig. 3c). The increase in reflectance ratio means that most of the incident THz waves are reflected on the surface.

Wrinkled structures can improve the conductivity of MXene films, thereby enhancing their impedance matching. The shielding behavior of MXene films against THz electromagnetic waves depends on the conductance of the film. Usually, films with high conductivity exhibit reflective dominant characteristics [51]. Time-domain and Frequency-domain spectra of the wrinkle-I film are shown in Fig. 3d. The THz transients are Fourier transformed to generate the transmission amplitude spectra. Compared with the PDMS substrate (named as Ref.), the transmitted THz signal of wrinkle-I film decreases significantly, indicating that the wrinkle-I film effectively blocks the transmission of terahertz electromagnetic waves. The THz field amplitude is significantly reduced as the thickness of the wrinkle-I film increases, as is expected by the increase in free carrier absorption (Fig. S8). In addition, based on the frequency-domain spectra (Figs. 3d and S10), we can use the Tinkham thin film equation to calculate the complex conductivity spectra of the MXene films [26], as shown in Fig. 3e. It can be seen that compared to flat film (about 330 Ω □^−1^), the wrinkle-I film shows a lower sheet resistance (about 200 Ω □^−1^).

Due to the unique inter-flakes charge transport characteristics of MXene, we found that the classical direct-current (DC) impedance theory is unsuitable for the mechanism analysis of MXene. However, the terahertz complex conductivity of the film can be well described by the Drude-Smith (DS) model, in which the free electrons transport is mainly in the form of oscillation and scattering under terahertz wave action [26]. The DS model equation is given as fellow:

1 $$\sigma \left(\omega \right)=\frac{{\sigma }_{0}}{1-i\omega \tau }\left(1+\frac{c}{1-i\omega \tau }\right)$$

where, $\sigma \left(\omega \right)$ is the conductivity of the film at different frequencies ($\omega$), as shown in Fig. 3e; ${\sigma }_{0}=\frac{N{e}^{2}\tau }{{m}^{*}}$ is the direct current (DC) conductivity; $N$,$\tau$,${m}^{*}$, and *c* are the charge carrier density, carrier scattering time, charge effective mass, and scattering parameter, respectively. The parameter *c* (-1 ≤ *c* ≤ 0) represents the scatting behaviors of the charges [26]. According to Eq. (1), the carrier scattering time $(\tau )$ of flat film and wrinkle-I film can be calculated to be 6.6 and 8.0 fs, respectively. The short carrier scattering time can meet the condition of *ωτ* ≪ 1 in the whole terahertz band, which proves that the dispersion of electrical conductivity becomes weak, and ensures both films can have strong absorption in the terahertz band. More importantly, the charge carrier density and surface density of electrons of the wrinkle-I film is 9.4 × 10^27^ and 7.5 × 10^19^ m^−2^, much higher than 6.0 × 10^27^ and 4.8 × 10^19^ m^−2^ of the flat film. The high electron concentration affects impedance matching, leading to higher terahertz absorption (R_□_ < Z_0_/2). According to the Woltersdoff equations, for a conducting thin film, the reflectance and transmittance ratio are negatively and positively related to the sheet resistance of the film, respectively (Fig. S11). However, both poor conductivity and good conductivity will lead to a low absorption. Only when the sheet resistance matches half of the free space resistance (Z_0_/2 = 188.5 Ω □^−1^), the thin film will show a maximum theoretical absorption limit of 50%. As shown in Fig. 3e, the sheet resistance of wrinkle-I MXene (200 Ω □^−1^) is closer to Z_0_/2, indicating that the introduction of the wrinkled structure improves the impedance matching of the thin film and enhances absorption for terahertz electromagnetic waves. In Fig. 3f, the equivalent alternating-current (AC) resistance collected at different frequencies agrees well with the theoretical calculations for the relevant frequency bands. Therefore, by improving the absorption performance of the wrinkle-I film in the broadband, it can approach the maximum EMI shielding in terahertz regions.

In addition, the surface of Ti_3_C_2_T_x_ nanosheets etched by the solution method usually contains a large number of functional groups (-O, -OH, -F) [52], making it difficult for free electrons to undergo boundary scattering and hopping transportation between nanosheets. Therefore, under electromagnetic wave irradiation, the free electron oscillations are completely bound to the film’ surface, which is known as surface plasmon polarization (SPP) [27]. The formation of wrinkled structures results in uneven distribution of free electrons on the surface of the MXene film, thereby enhancing local SPP. The SPP can enhance the interaction and absorption of terahertz electromagnetic waves in the films. Similar phenomena have been extensively observed in far-infrared light [53]. Therefore, the wrinkled structure in the MXene film improved the EMI SE for THz waves.

### Stretchability and Stability of THz EMI Performance of the Wrinkled Films

**Fig. 4:**
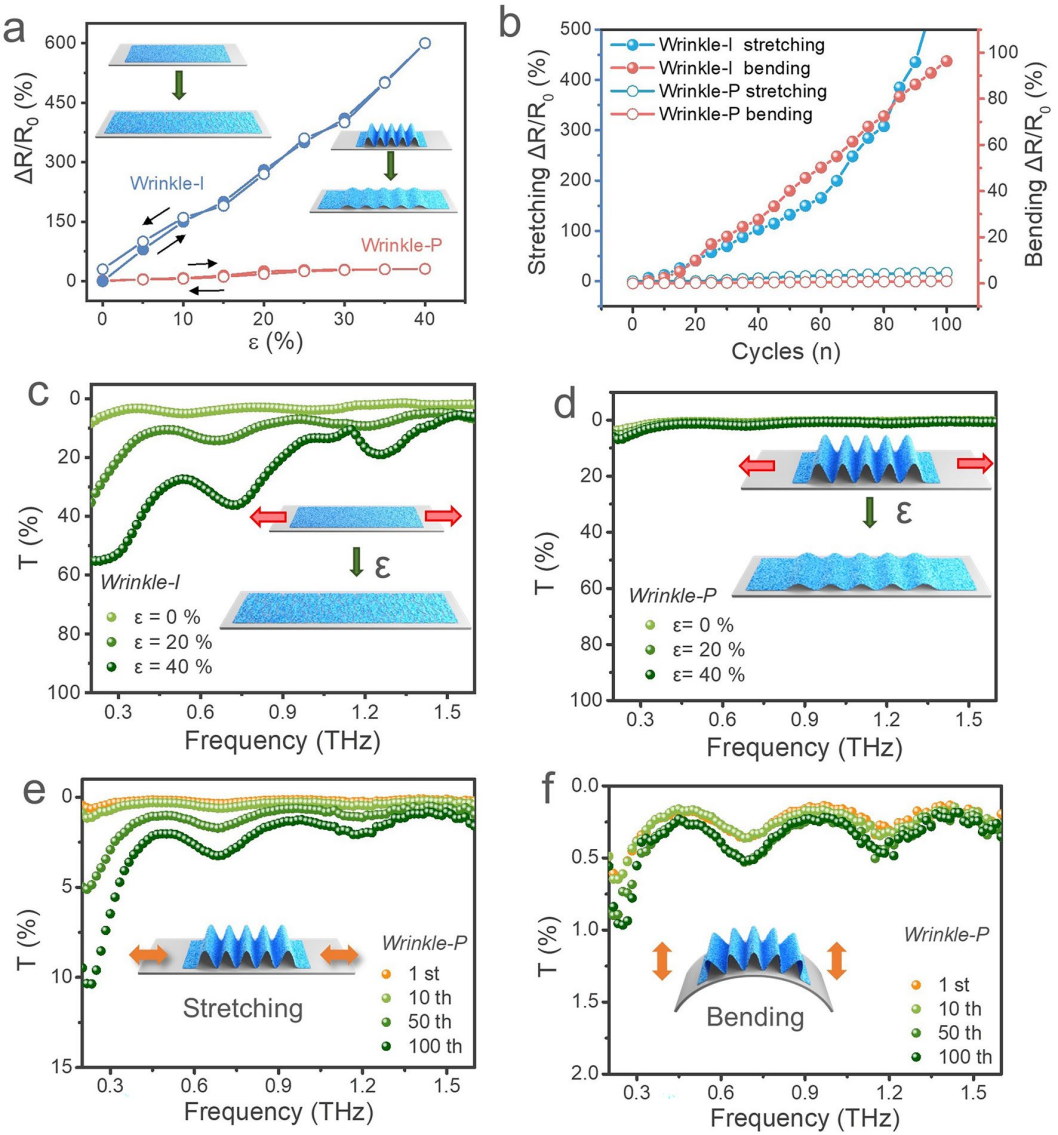
Stretchability and stability of electromagnetic shielding performance of the wrinkled film. The wrinkle-P film was fabricated by PDMS substrate with pre-stretching strain of 40%. **a** Relative resistance changes of the wrinkled films during continuous loading and unloading processes. **b** Relative resistance changes of the wrinkled films under cyclic stretching (left ordinate) and bending (right ordinate). Transmittance of the wrinkle-I film **c**, and wrinkle-P film **d** under different tensile strains. Transmittance of the wrinkle-P film under different cycles of **e** stretching test with a strain of 30%, and **f** bending test with a bending radius of 8 mm

To achieve stretchable terahertz EMI shielding film, longitudinal periodic wrinkles were introduced to the MXene by releasing the pre-stretched PDMS substrate during the same self-assembly process. The microstructures of wrinkle-P films prepared under different pre-stretched stains are shown in Fig. S12. The periodic wrinkles can effectively accommodate the high degree of deformation with little impact on the conductivity, significantly enhancing the stretchability and resistance stability of the wrinkled MXene film. Consequently, the wrinkle-P film with longitudinal wrinkles exhibited a remarkable resistance stability with only 31.1% resistance increase even under a high tensile strain of about 40%. In comparison, the value was 600.0% for the wrinkle-I film (Fig. 4a). The outstanding strain-independent conductivity will allow the wrinkle-P film to withstand the dynamic deformation (compression and tension) without impacting the shielding performance. Moreover, the resistance variations (△*R*/*R*_0_) of the wrinkle-P film can return to the starting point without detectable conductivity decline, indicating that longitudinal wrinkles benefit the stretchability and prevent irreversibly damaging the conductivity. We conducted stretching (up to 30% strain) and bending (with a bending radius of 8 mm) fatigue tests for 100 cycles to evaluate the robustness of the films. As shown in Fig. 4b, the resistances of the wrinkle-P film just increased by 16.8% after 100 stretching-releasing cycles, which is much lower than those of the wrinkle-I film (△*R*/*R*_0_ > 500%). The wrinkle-P film exhibits excellent stability and robustness in dynamic environments, which makes it a promising candidate for conformal shielding materials in wearable electronics.

As shown above, the wrinkle-P film has outstanding resistance stability, which ensures its stable shielding performance. Figure 4c, d show that the wrinkle-P film has a lower THz transmittance value when compared with the wrinkle-I film. This indicates that the longitudinal wrinkles can improve the EMI SE of the MXene film. The transmittance of the wrinkle-P film only increased by 0.8% (SE decreased by 3.3 dB) within the THz band as the strain increases from 0 to 40% (Fig. 4d). However, the THz transmittance of the wrinkle-I film dramatically increased from 3.0% (SE of 15.2 dB) to 24.5% (SE of 6.1 dB) with the same strain range (Fig. 4c). Similar results were also observed for the wrinkle-P film prepared under different pre-stretching strains (Fig. S13). The wrinkle-I has a significant decline in EMI SE due to the fractures generated during the stretching process (Fig. S14a-c). In contrast, the longitudinal wrinkles effectively prevent the fractures and cracks forming on the MXene film, thereby ensuring the stability of the EMI SE (Fig. S14d-f). Combining the THz transmittance results and SEM images, it can be concluded that the longitudinal wrinkles are beneficial in suppressing the attenuation of EMI SE during stretching process. Constructing wrinkled structures is one of the effective ways to achieve strain-invariant EMI shielding film.

The stability of multiple stretching/bending behaviors is the most basic requirement of flexible electronic devices, so it is necessary to analyze the mechanical stability of the wrinkled film. The wrinkle-P and wrinkle-I films were stretched or bent for 100 cycles at different strains, recording the THz transmittance after specific cycles (1st, 10th, 50th, and 100th cycle). As shown in Fig. 4e, the average THz transmittance of the wrinkle-P film, fabricated using PDMS substrate pre-stretched in 40%, is only 1.2% and 2.4% after 50 and 100 cycles at 30% strain, respectively. However, for the wrinkle-I film, the average THz transmittance has exceeded 55.6% after 100 cycles at 30% strain (Figs. S15 and S16). The THz transmittance of the wrinkle-P film has hardly increased, remaining around 0.5% during the bending test (Fig. 4f). Correspondingly, it increased to 3.0% after 100 cycles for the wrinkle-I film (Fig. S17). These results indicate that the wrinkled-P structure is beneficial for improving the film’s mechanical properties, thereby enhancing its stability in electromagnetic shielding performance during deformation, such as stretching/bending.

**Fig. 5:**
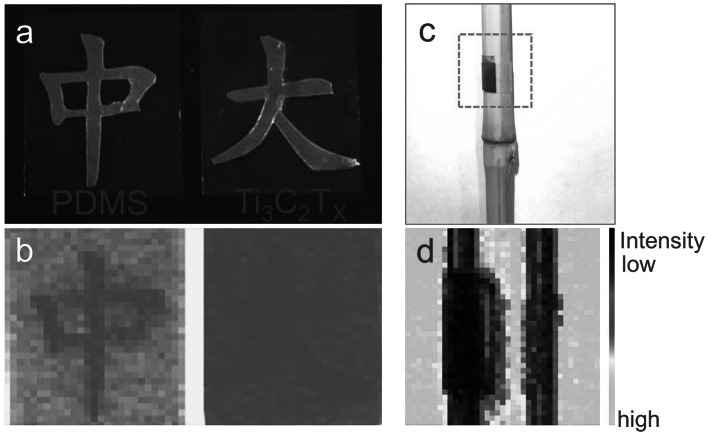
Electromagnetic interference shielding application of the wrinkled film in THz imaging. **a** Optical photos, and **b** THz imaging photos of an aluminum foil in the shape of Chinese characters of "中" and "大" covered with a PDMS film (left) and wrinkle-I film (right), respectively. **c** Optical photo, and **d** THz imaging photo of a wrinkle-P film attached to the surface of a dried bamboo. It demonstrates the transparency, conformability, and THz shielding effect of the film

Compared with conventional rigid materials, wrinkled films can better cover irregular surfaces. The wrinkled films were used to shield THz electromagnetic waves during THz imaging and effectively demonstrate its excellent EMI SE and conformal properties. A PDMS film and an 8-nm-thick wrinkle-I film were placed on the surface aluminum foil in the shape of Chinese characters of "中" and "大", respectively. Both the PDMS and wrinkled film deliver high light transmittance, allowing Chinese characters "中" and "大" to be clearly observed (Fig. 5a). Figure 5b is the THz imaging result of Fig. 5a. The winkled film can effectively shield terahertz waves, resulting in the disappeared "大" in the THz imaging. Similarly, the wrinkled-P film was attached to a dried- bamboo surface to verify its transparency, conformability, and THz shielding effect (Fig. 5c). The THz imaging results demonstrate that the winkled film coverage area has the lowest terahertz wave transmission intensity (deep purple area), indicating that the film can still maintain excellent terahertz shielding performance in the bent state.

## Conclusion

We have proposed a structure-engineering strategy to prepare stretchable and transparent Ti_3_C_2_T_x_ film with controllably wrinkled structures for terahertz EMI shielding. The wrinkled structure on the surface of the MXene film enhances the absorption of terahertz waves by improving impedance matching characteristics and enhancing local SPP, which further enhances the EMI SE of the wrinkled film. Compared with the flat film, the average EMI SE value of the 8 nm thick wrinkle-I film is increased by 36.5% over a wide frequency range of 0.1–10 THz. Furthermore, the wrinkle-P film exhibits a low THz transmittance of only increased by 0.8% under 40% strain and also excellent structural stability under multiple stretching/bending processes. The electromagnetic shielding effect of the wrinkled films also has been verified in real-object THz imaging. This new strategy may pave the way for exploring practical high-performance THz electromagnetic wave shielding films based on two-dimensional materials.

## Supplementary Information

Below is the link to the electronic supplementary material.

Supplementary file1 (PDF 869 KB)
