# Supplementary material for: Stretchable, Transparent, and Ultra-Broadband Terahertz Shielding Thin Films Based on Wrinkled MXene Architectures
Source: Nanomicro Lett. 2024 Apr 2;16:165. doi: 10.1007/s40820-024-01365-w (PMC10987438; doi:10.1007/s40820-024-01365-w)
Supplement: Supplementary file 1 — Supplementary file1 (PDF 869 KB) [file 40820_2024_1365_MOESM1_ESM.pdf]

Supporting Information for

Stretchable, Transparent, and Ultra-Broadband Terahertz Shielding Thin Films Based on Wrinkled MXene Architectures

Shaodian Yang[1,†], Zhiqiang Lin[2,†], Ximiao Wang[1,3,†], Junhua Huang[1], Rongliang Yang[1], Zibo Chen[1], Yi Jia[4], Zhiping Zeng[5], Zhaolong Cao[1,3], Hongjia Zhu[1,3], Yougen Hu[2], Enen Li[6,7,8], Huanjun Chen[1,3,*], Tianwu Wang[6,7,8,*], Shaozhi Deng[1,3,*], and Xuchun Gui[1,*]

[1]State Key Laboratory of Optoelectronic Materials and Technologies, School of Electronics and Information Technology, Sun Yat-sen University, Guangzhou 510275, P. R. China

[2]National Key Laboratory of Materials for Integrated Circuits, Shenzhen Institute of Advanced Electronic Materials, Shenzhen Institute of Advanced Technology, Chinese Academy of Sciences, Shenzhen 518055, P. R. China

[3]Guangdong Province Key Laboratory of Display Material and Technology, Guangzhou 510275, P. R. China

[4]China Academy of Aerospace Science and Innovation, Beijing 100176, P. R. China

[5]School of Materials Science and Engineering, Sun Yat-sen University, Guangzhou 510275, P. R. China

[6]GBA Branch of Aerospace Information Research Institute, Chinese Academy of Sciences, Guangzhou 510700, P. R. China

[7]School of Electronic, Electrical and Communication Engineering, University of Chinese Academy of Sciences, Beijing 100049, P. R. China

[8]Guangdong Provincial Key Laboratory of Terahertz Quantum Electromagnetics, Guangzhou 510700, P. R. China

[†] Shaodian Yang, Zhiqiang Lin, and Ximiao Wang contributed equally.

*Corresponding authors. E-mail: guixch@mail.sysu.edu.cn (Xuchun Gui), wangtw@aircas.ac.cn (Tianwu Wang), chenhj8@mail.sysu.edu.cn (Huanjun Chen), stsdsz@mail.sysu.edu.cn (Shaozhi Deng)

S1 THz Conductivity of MXene Films Measured by THz TDS System

The terahertz EMI shielding property (in 0.2-1.6 THz) was obtained using a fiber-coupled terahertz time-domain spectroscopy (THz-TDS) system in transmission mode. Broadband terahertz spectroscopy up to 10 THz was performed using two-color laser induced air plasma terahertz generation system combined with air biased coherent detection method. The sample size is 10 ×10 mm^2. Through THz-TDS, we can directly obtain the electric field amplitudes (intensities) of the transmitted signal as a function

of time for the samples with substrate ($E_{sam}(t)$), and substrate ($E_{sub}(t)$). Furthermore, utilizing Fourier transform, we can obtain the amplitudes as a function of frequency (ω) for the samples with substrate ($\tilde{E}_{sam}(\omega)$), and substrate ($\tilde{E}_{sub}(\omega)$). Therefore, the transmissivity (T) of the samples was determined by:

$$SE_T = -10\ logT = -20log\frac{\tilde{E}_{sam}(\omega)}{\tilde{E}_{sub}\ (\omega)} \qquad \text{(S1)}$$

According Tinkham film equation, the complex conductivity spectrum ($\tilde{\sigma}(\omega)$) of the MXene film can be extracted by:

$$\frac{\tilde{E}_{sam}(\omega)}{\tilde{E}_{sub}\ (\omega)} = \frac{n_s+1}{n_s+1+Z_0 d\tilde{\sigma}(\omega)} \qquad \text{(S2)}$$

Where, n_s = 1.41 is the index of substrate (PDMS in the article), Z_0= 377 Ω is the free space impedance, d is the film thickness.

According to Fresnel's formula, using the transmission and reflection coefficients, the transmission and reflection coefficients can be obtained as: [S1]

$$T = \left|\frac{2\sqrt{n_0 n_s}}{n_0+n_s+Z_0\tilde{\sigma}(\omega)}\right|^2 \qquad \text{(S3)}$$

$$R = \left|\frac{n_0-n_s-Z_0\tilde{\sigma}(\omega)}{n_0+n_s+Z_0\tilde{\sigma}(\omega)}\right|^2 \qquad \text{(S4)}$$

where n_0=1 is the air refractive index.

Therefore, the absorption can be obtained as:

$$A = 1 - T - R \qquad \text{(S5)}$$

S2 Fitting of THz Conductivity with the Drude-Smith Model

The transport behavior of free carriers can be well described by the Drude-Smith (DS) model:

$$\sigma(\omega) = \frac{\sigma_0}{1-i w\tau}\left(1 + \frac{c}{1-i w\tau}\right) \qquad \text{(S6)}$$

where $\sigma_0 = \frac{Ne^2\tau}{m^*}$ is the direct current (DC) conductivity; N, τ, m^*, and c are the charge carrier density, carrier scattering time, charge effective mass, and scattering parameter, respectively. The parameters of τ, N, and c were obtained by fitting the measured conductivity in Fig. 3e with the DS model. The fitted parameters are listed in Table S2.

Table S2 Fitting parameters using the Drude-Smith model

	τ (fs)	c	N (m^{-3})	σ_0 (mS)
Flat	6.6	-0.75	6.0×10^{27}	8.9
Wrinkle-I	8.0	-0.75	9.4×10^{27}	16.9

S3 The Impedance Calculation

The sheet resistance-dependent relationship between the transmittance (*T*), reflection (*R*), and absorption (*A*) of a thin film are given as follows.

$$T = \frac{4g^2}{(1+2g)^2} \tag{S7}$$

$$R = \frac{1}{(1+2g)^2} \tag{S8}$$

$$A = \frac{4g}{(1+2g)^2} \tag{S9}$$

$$g = R_{\square}/Z_0 \tag{S10}$$

where $R_{\square}$ is the sheet resistance of the film, Z_0 is the impedance of free space, which is equal to 377 Ω.

S4 Supplementary Figures

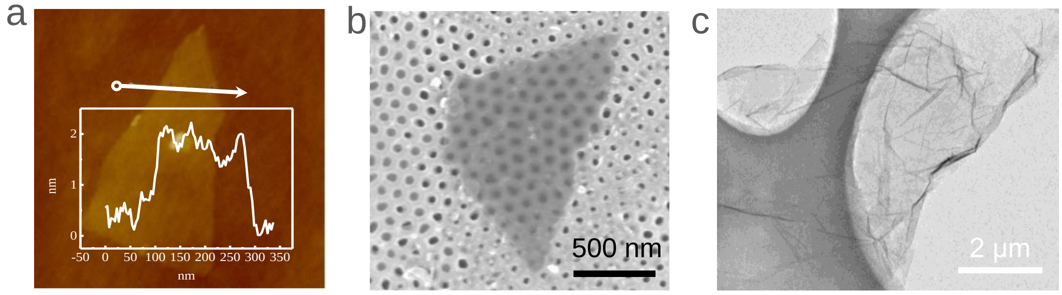

Fig. S1 a AFM image, **b** SEM image, and **c** TEM image of $Ti_3C_2T_x$ nanosheets

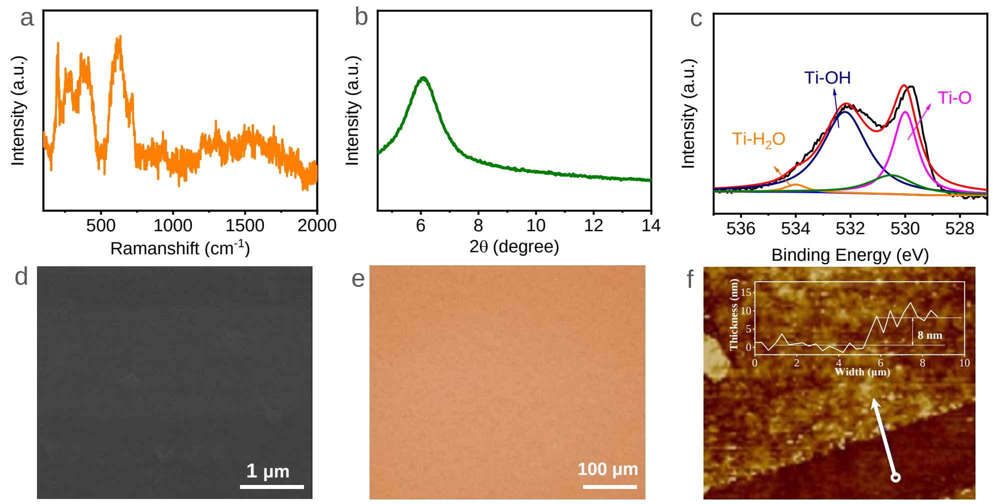

Fig. S2 a Raman spectrum, **b** XRD pattern, **c** XPS spectra of O 1s, **d** SEM image, **e** optical image, and **f** AFM image of the transparent flat film. Insert of **f** is the thickness profile of the flat film

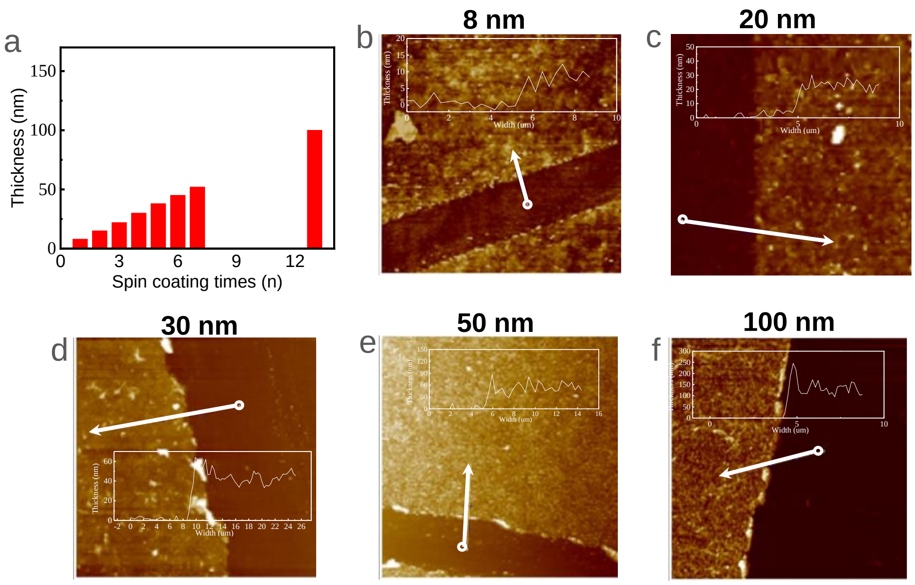

Fig. S3 a Relationship of thickness versus spin coating time in $Ti_3C_2T_x$ MXene films. **b-f** AFM images of the $Ti_3C_2T_x$ films with different thicknesses

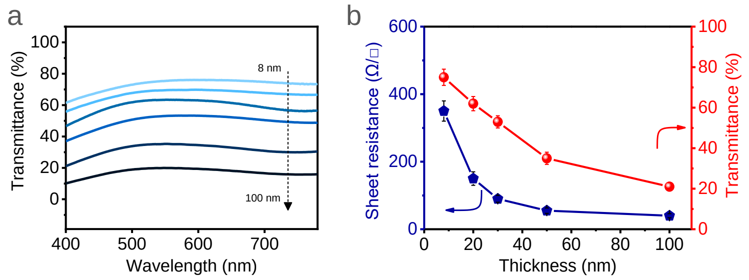

Fig. S4 a UV-vis transmittance spectra of flat $Ti_3C_2T_x$ films with different thicknesses. **b** Square resistances and light transmittances of the flat films

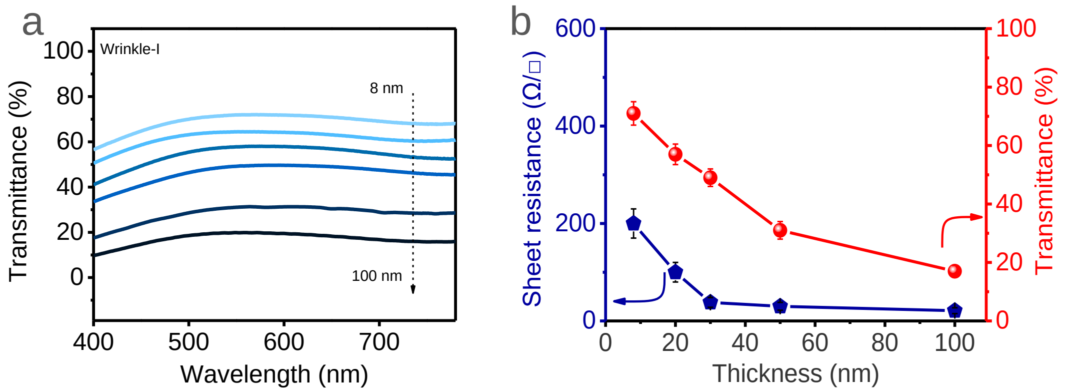

Fig. S5 a UV-vis transmittance spectra of wrinkle-I films with different thicknesses. **b** Square resistances and light transmittances of the wrinkle-I films

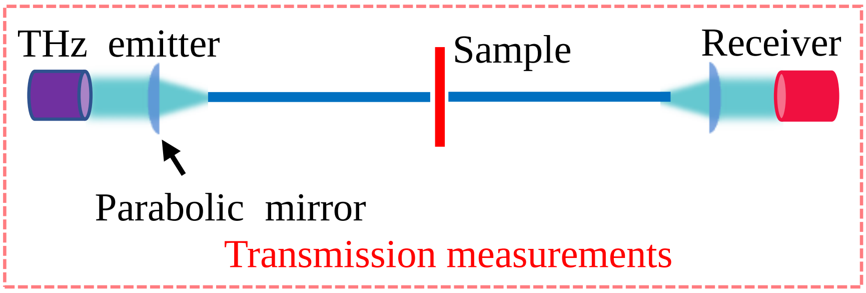

Fig. S6 Schematic illustration of the THz-TDS system for the transmission measurements. The THz time-domain spectroscopy (THz-TDS) system is mainly composed of the femtosecond laser, emitter, detector, and time delay device. The femtosecond laser is divided into two channels through the beam splitter: one channel is directly incident through the optical path to the emitter to achieve terahertz radiation; The other channel passes through the time delay device and then incident into the detector to achieve terahertz detection. Terahertz imaging also uses the same TDS-THz system, which place an automatic displacement stage between the transmit and receive ports. The test sample (shielding film) is first fixed on the displacement stage, and the position of the sample can be changed by controlling the movement of the stage. After collecting shielding effects at each point of the sample, the terahertz imaging of shielding film was plotted. Each pixel of terahertz imaging in this manuscript is 2×2 mm^2.

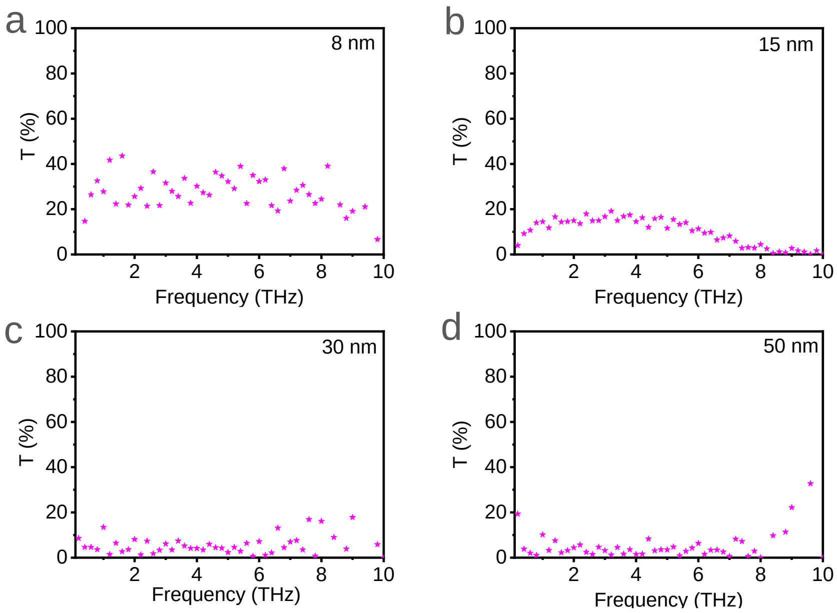

Fig. S7 Terahertz wave transmittances (0.1-10 THz) of the wrinkle-I films with thicknesses of **a** 8 nm, **b** 15 nm, **c** 30 nm, and **d** 50 nm. The terahertz wave (0.1-10 THz) transmittance of the wrinkled film also reduced with its thickness. When the thickness of the wrinkle-I film is more than 30 nm, its terahertz wave transmittance can be less than 10%, and the corresponding EMI SE is greater than 10 dB

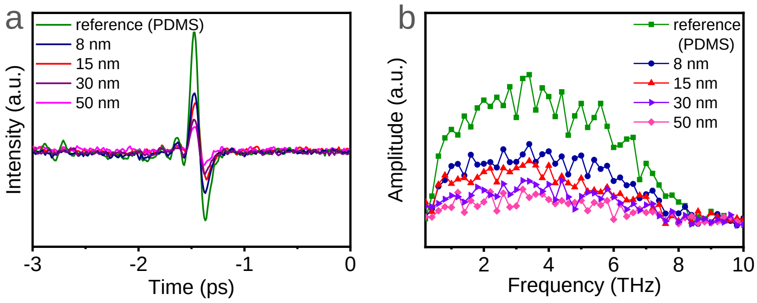

Fig. S8 **a** Time-domain spectra, and **b** Frequency-domain spectra of the wrinkle-I film with different thicknesses. Compared with the pristine signal of PDMS, the transmitted THz signal of wrinkle-I film decreases rapidly with the increase of $Ti_3C_2T_x$ thickness.

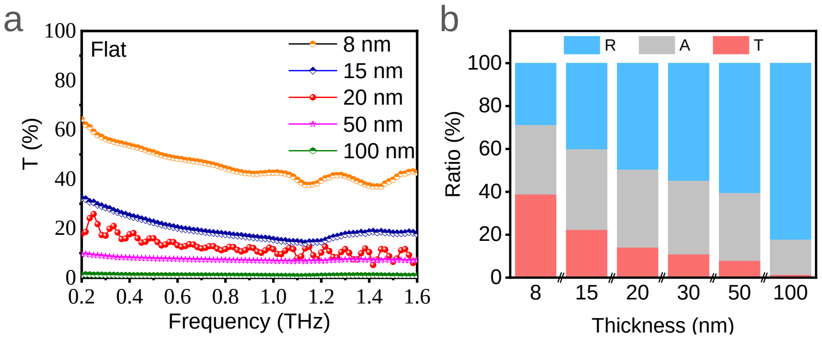

Fig. S9 **a** THz transmittances of the flat film with different thicknesses. **b** Ratio of THz transmittance, reflection, and absorption of the flat film with different thicknesses

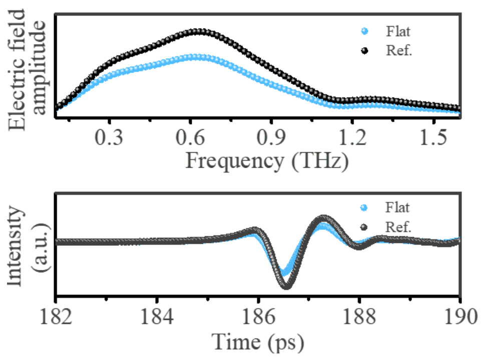

Fig. S10 Transmission amplitude spectra (up) of the flat film, obtained by Fourier transforming the transients (down)

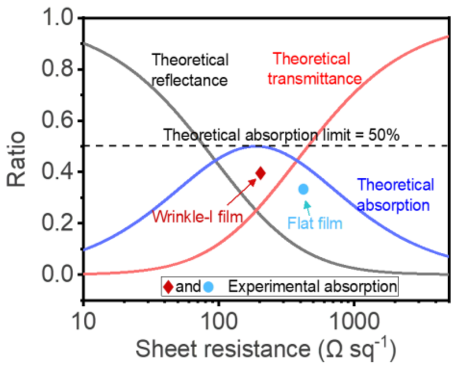

Fig. S11 Theoretical and experimental comparison of THz absorption between flat film and wrinkle-I film

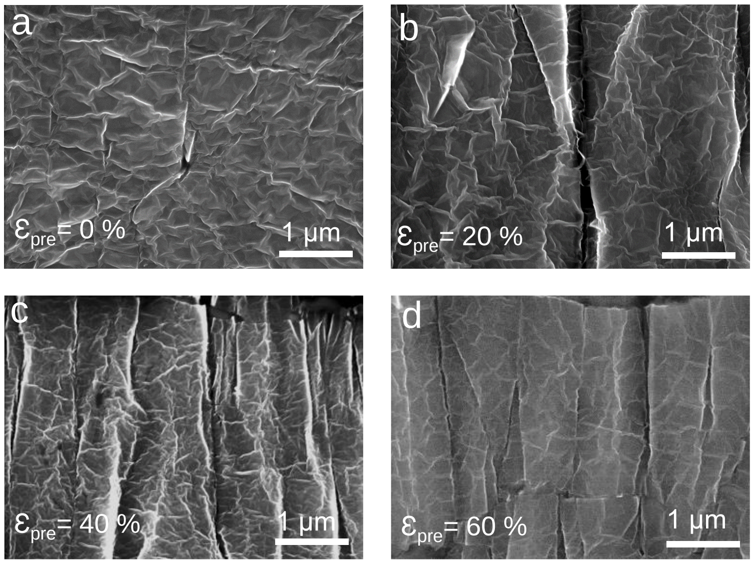

Fig. S12 SEM images of the wrinkle-I film and wrinkle-P film, which fabricated by PDMS substrate with different pre-stretching strains (ε_{pre}). **a** wrinkle-I film (ε_{pre}= 0%), **b** wrinkle-P film (ε_{pre}= 20%), **c** wrinkle-P film (ε_{pre}= 40%), and **d** wrinkle-P film (ε_{pre}= 60%). The wrinkle-P films with different pre-stretching strains have both isotropic and periodic wrinkles. In addition, the heights and densities of periodic wrinkles also increased with their thickness.

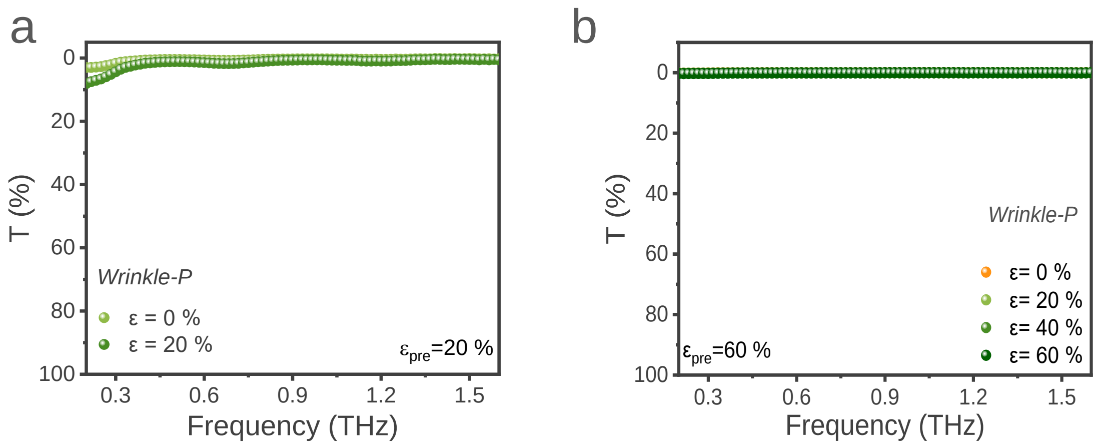

Fig. S13 THz transmittances of the film under different tensile strain. **a** wrinkle-P film (ε_{pre}= 20%), and **b** wrinkle-P film (ε_{pre}= 60%). It demonstrated that all wrinkle-P films are provided with excellent stretching stability

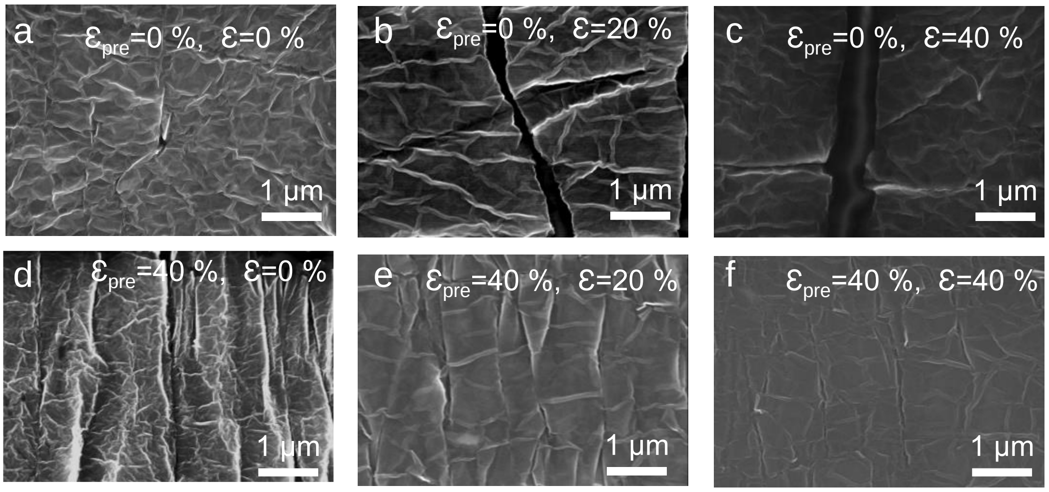

Fig. S14 SEM images of wrinkle-I film (ε_{pre}= 0%) under different tensile strains **a** 0%, **b** 20%, and **c** 40%. SEM images of wrinkle-P film (ε_{pre}= 40%) under different tensile strains **d** 0%, **e** 20%, and **f** 40%

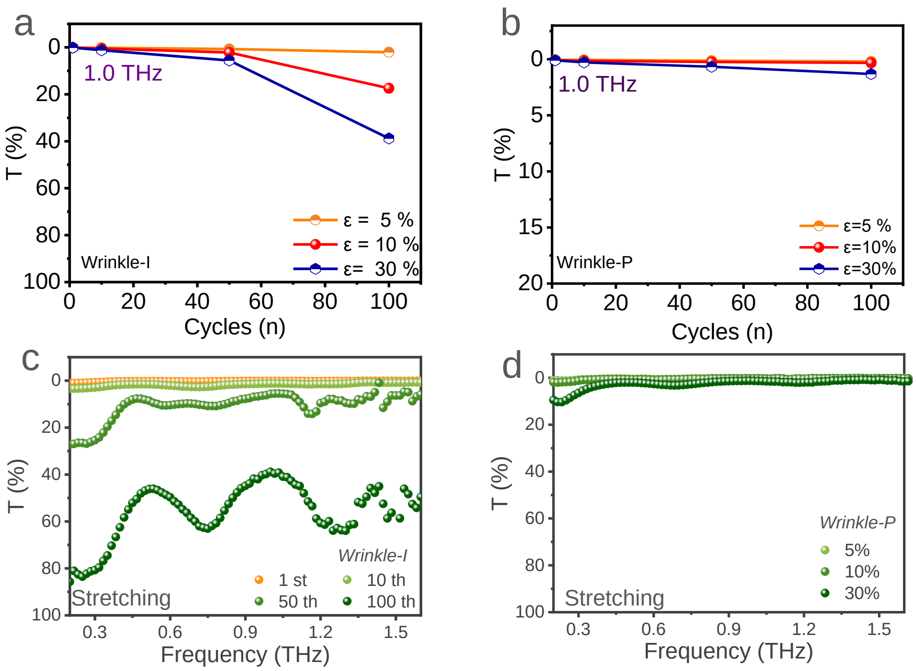

Fig. S15 a THz transmittances of wrinkle-I film, and **b** wrinkle-P film under various strains (5%, 10%, 30%) at the different cycles (0, 10th,50th,100th) in 1.0 THz. **c** THz transmittances of wrinkle-I film during the 100-cycle stretching test with 30% strain. **d** THz transmittances of wrinkle-P film after the 100-cycle stretching test with various strains in the frequency of 0.2-1.6 THz. The wrinkle-P film was fabricated by PDMS substrate with pre-stretching strain of 40%

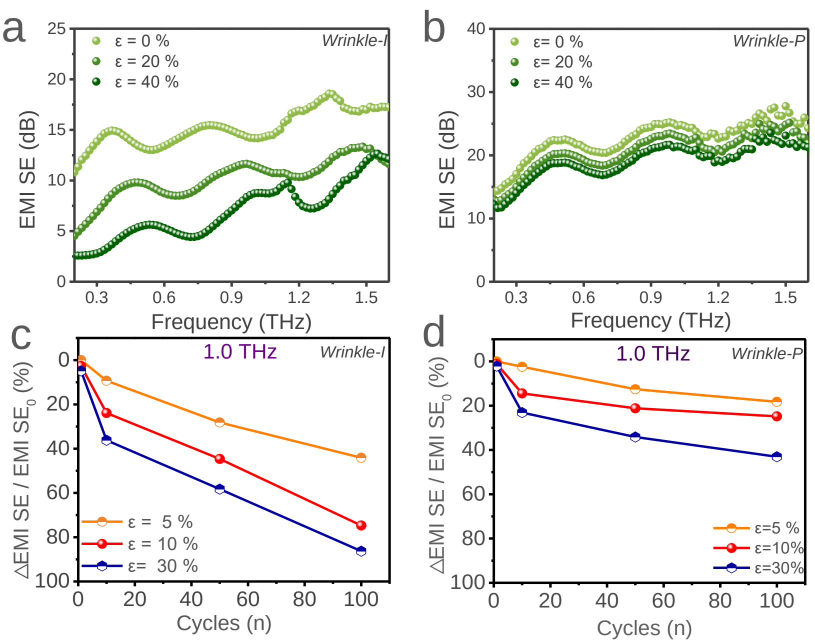

Fig. S16 The EMI SE values of **a** wrinkle-I film, and **b** wrinkle-P film under different stretching strains (0-40%). **c** The △EMI SE / EMI SE_0 values of wrinkle-I film, and **d** wrinkle-P film under various strains (5%, 10%, 30%) at the different cycles (0th, 10th, 50th, 100th) at 1.0 THz

The wrinkle-P film was fabricated by PDMS substrate with pre-stretching strain of 40%. In **c** and **d**, EMI SE_0 is the electromagnetic shielding effectiveness of the sample before stretching test. EMI SE_n is the electromagnetic shielding effectiveness of the sample after *n* cycles of stretching under a specific strain. △EMI SE = EMI SE_0 – EMI SE_n.

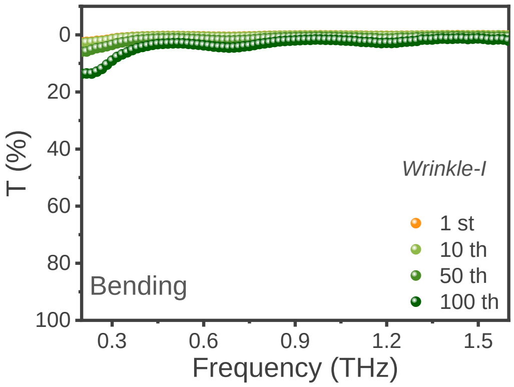

Fig. S17 THz transmittances of wrinkle-I film during the 100-cycle bending test with the bending radius of 8 mm in the frequency of 0.2-1.6 THz

Table S1 Thickness, EMI shielding performance, thickness averaged specific EMI SE and frequency range of various shielding materials

Sample	Thickness (μm)	EMI (dB)	SE/t (dB $μm^{-1}$)	Frequency range (Hz)	Refs.
MGF	3000	61	0.020	0.2-1.6 T	[S2]
$Ti_3C_2T_X$ -PAA-ACC	130	45	0.346	0.2-2.0 T	[S3]
rGO	370	65	0.175	0.1-1.0 T	[S4]
MSF	10000	45	0.004	0.3-1.6 T	[S2]
Cu foil	0.04	44	1100	0.1-1.0 T	[S5]
	10	70	7	8.2-12.4 G	[S6]
Al foil	8	68	8.5	8.2-12.4 G	[S6]
Ti_3CNT_X	40	116.2	2.9	8.2-12.4 G	[S7]
$Ti_3C_2T_X$	40	93	2.7	8.2-12.4 G	[S7]
Ni/PVDF	500	35.4	0.7	18-26.5 G	[S8]
Silver Nanowire	0.1	8	80	0-2.5 T	[S9]
graphene/PMMA	6	15	2.5	0.2-2.0 T	[S10]
MXene/rGO film	148	54.2	0.366	0.2-2.0 T	[S11]
CNT	0.01	8	80	0.2-2.5T	[S12]
PAL-$Ti_3C_2T_X$	38.3	50.5	1.318	0.2-1.6 T	[13]
Zn^{2+}-$Ti_3C_2T_X$	85	51	0.6	0.2-2.0 T	[S14]
Carbon	125	65	0.52	0.22-0.5 T	[S15]
Cu-Ag-ITO	50	26	0.52	8.2-40.0 G	[S16]
Ag NC thin film	1.3	60	48	8.2-12.4 G	[S17]

MXene-based fiber	213	83.4	0.39	8.2-12.4 G	[S18]
MXene-Based fiber	370	48	0.13	8.2-12.4 G	[S19]
PVA hydrogel	3000	62.5	0.02	8.2-12.4 G	[S20]
CNT-based	2000	113	0.06	8.2-12.4 G	[S21]
$Ti_3C_2T_x$/CNF aerogels	2000	79	0.04	8.2-12.4 G	[S22]
$Ti_3C_2T_x$-based	75	85	1.13	8.2-12.4 G	[S23]
Biocarbon nano paper	600	46	0.08	0.4-2.0 T	[S24]
CNT-MXene film	3000	48.2	0.06	0.1-2.2 T	[S25]
Transparent $Ti_3C_2T_X$	0.008	5.6	700	0.2-10.0 T	This work

Supplementary References

[S1] Haus, Hermann A., Waves and Fields in Optoelectronics (Prentice-Hall Series in Solid State Physical Electronics). Englewood Cliffs, New Jersey: Prentice Hall, 1984.SERIES IN SOLID STATEPHYSICAL ELECTRONICS, 1984).

[S2] W. Shui, J. Li, H. Wang, Y. Xing, Y. Li et al., $Ti_3C_2T_X$ MXene sponge composite as broadband terahertz absorber. Adv. Opt. Mater. **8**, 2001120 (2020). https://doi.org/10.1002/adom.202001120

[S3] Y. Zhu, J. Liu, T. Guo, J. J. Wang, X. Tang et al., Multifunctional $Ti_3C_2T_X$ MXene composite hydrogels with strain sensitivity toward absorption-dominated electromagnetic-interference shielding. ACS Nano **15**, 1465 (2021). https://doi.org/10.1021/acsnano.0c08830

[S4] S. Dong, Q. Shi, W. Huang, L. Jiang, and Y. Cai, Flexible reduced graphene oxide paper with excellent electromagnetic interference shielding for terahertz wave. J. Mater. Sci. Mater. Electron. **29**, 17245 (2018). https://doi.org/10.1007/s10854-018-9818-1

[S5] S. Hou, W. Ma, G. Li, Y. Zhang, Y. Ji et al., Excellent terahertz shielding performance of ultrathin flexible Cu/graphene nanolayered composites with high stability. J. Mater. Sci. Technol. **52**, 136 (2020). https://doi.org/10.1016/j.jmst.2020.04.007

[S6] F. Shahzad, M. Alhabeb, C. B. Hatter, B. Anasori, S. Man Hong et al., Electromagnetic interference shielding with 2D transition metal carbides (MXenes). Science **353**, 1137 (2016). https://doi.org/10.1126/science.aag2421

[S7] Y. Zhou, M. Wu, J. Jiang, P. Yang, T. Rao et al., Self-assembling SiC nanoflakes/MXenes composites embedded in polymers towards efficient electromagnetic wave attenuation. Appl. Surf. Sci. **574**, 151463 (2022). https://doi.org/10.1016/j.apsusc.2021.151463

[S8] B. Zhao and C. B. Park, Tunable electromagnetic shielding properties of conductive poly(vinylidene fluoride)/Ni chain composite films with negative permittivity. J. Mater. Chem. C **5**, 6954 (2017).

https://doi.org/10.1039/c7tc01865g

[S9] N. Hoof, M. Parente, A. Baldi, and J. G. Rivas, Terahertz time-domain spectroscopy and near-field microscopy of transparent silver nanowire networks. Adv. Opt. Mater. **8**, 1900790 (2020). https://doi.org/10.1002/adom.201900790

[S10] C. Pavlou, M. G. Pastore Carbone, A. C. Manikas, G. Trakakis, C. Koral et al., Effective emi shielding behaviour of thin graphene/pmma nanolaminates in the thz range. Nat. Commun. **12**, 4655 (2021). https://doi.org/10.1038/s41467-021-24970-4

[S11] S. Li, S. Xu, K. Pan, J. Du, and J. Qiu, Ultra-thin broadband terahertz absorption and electromagnetic shielding properties of MXene/rGO composite film. Carbon N. Y. **194**, 127 (2022). https://doi.org/10.1016/j.carbon.2022.03.048

[S12] M. A. Seo, J. H. Yim, Y. H. Ahn, F. Rotermund, D. S. Kim et al., Terahertz electromagnetic interference shielding using single-walled carbon nanotube flexible films. Appl. Phys. Lett. **93**, 231905 (2008). https://doi.org/10.1063/1.3046126

[S13] T. Yun, H. Kim, A. Iqbal, Y. S. Cho, G. S. Lee et al., Electromagnetic shielding of monolayer MXene assemblies. Adv. Mater. **32**, 1906769 (2020). https://doi.org/10.1002/adma.201906769

[S14] Z. Lin, J. Liu, W. Peng, Y. Zhu, Y. Zhao et al., Highly stable 3D $Ti_3C_2T_X$ MXene-based foam architectures toward high-performance terahertz radiation shielding. ACS Nano **14**, 2109 (2020). https://doi.org/10.1021/acsnano.9b08832

[S15] S. Venkatachalam, D. Bertin, G. Ducournau, J. F. Lampin, and D. Hourlier, Kapton-derived carbon as efficient terahertz absorbers. Carbon **100**, 158 (2016). https://doi.org/10.1016/j.carbon.2016.01.003

[S16] J. Jung, H. Lee, I. Ha, H. Cho, K. K. Kim et al., Highly stretchable and transparent electromagnetic interference shielding film based on silver nanowire percolation network for wearable electronics applications. ACS Appl. Mater. Interfaces **9**, 44609 (2017). https://doi.org/10.1021/acsami.7b14626

[S17] S. Park, J. Bang, B. S. Kim, S. J. Oh, and J. H. Choi, Metallic fusion of nanocrystal thin films for flexible and high-performance electromagnetic interference shielding materials. Mater. Today Adv. **12**, 100177 (2021). https://doi.org/10.1016/j.mtadv.2021.100177

[S18] L. X. Liu, W. Chen, H. Bin Zhang, L. Ye, Z. Wang et al., Super-tough and environmentally stable aramid. nanofiber@MXene coaxial fibers with outstanding electromagnetic interference shielding efficiency. Nano-Micro Lett. **14**, 111 (2022). https://doi.org/10.1007/s40820-022-00853-1

[S19] J. Wang, X. Ma, J. Zhou, F. Du, and C. Teng, Bioinspired, high-strength, and flexible MXene/aramid fiber for electromagnetic interference shielding papers with joule heating performance. ACS Nano **16**, 6700 (2022).

https://doi.org/10.1021/acsnano.2c01323

[S20] B. Zhao, Z. Bai, H. Lv, Z. Yan, Y. Du et al., Self-healing liquid metal magnetic hydrogels for smart feedback sensors and high-performance electromagnetic shielding. Nano-Micro Lett. **15**, 79 (2023). https://doi.org/10.1007/s40820-023-01043-3

[S21] J. Li, H. Sun, S. Q. Yi, K. K. Zou, D. Zhang et al., Flexible polydimethylsiloxane composite with multi-scale conductive network for ultra-strong electromagnetic interference protection. Nano-Micro Lett. **15**, 15 (2023). https://doi.org/10.1007/s40820-022-00990-7

[S22] L. Wang, Z. Ma, H. Qiu, Y. Zhang, Z. Yu et al., Significantly enhanced electromagnetic interference shielding performances of epoxy nanocomposites with long-range aligned lamellar structures. Nano-Micro Lett. **14**, 224 (2022). https://doi.org/10.1007/s40820-022-00949-8 .

[S23] Y. Zhang, K. Ruan, K. Zhou, and J. Gu, Controlled distributed $Ti_3C_2T_X$ hollow microspheres on thermally conductive polyimide composite films for excellent electromagnetic interference shielding. **35**, 2211642 (2023). https://doi.org/10.1002/adma.202211642

[S24] A. R. Pai, Y. Lu, S. Joseph, N. M. Santhosh, R. Degl'Innocenti et al., Ultra-broadband shielding of cellulose nanofiber commingled biocarbon functional constructs: a paradigm shift towards sustainable terahertz absorbers. Chem. Eng. J. **467**, 143213 (2023). https://doi.org/10.1016/j.cej.2023.143213

[S25] [S25] Y. Guo, Y. Bai, and Y. Lu, Flexible CNT-MXene-CNT film with low surface conductance for high-and low-power electromagnetic absorption protection. ACS Appl. Electron. Mater. **5**, 6859 (2023). https://doi.org/10.1021/acsaelm.3c01285
